# Structural determinants for protein unfolding and translocation by the Hsp104 protein disaggregase

**DOI:** 10.1042/BSR20171399

**Published:** 2017-12-22

**Authors:** Jungsoon Lee, Nuri Sung, Lythou Yeo, Changsoo Chang, Sukyeong Lee, Francis T.F. Tsai

**Affiliations:** 1 Verna and Marrs McLean Department of Biochemistry and Molecular Biology, Baylor College of Medicine, One Baylor Plaza, Houston, TX 77030, U.S.A.; 2 Department of Molecular and Cellular Biology, Baylor College of Medicine, One Baylor Plaza, Houston, TX 77030, U.S.A.; 3 Department of Molecular Virology and Microbiology, Baylor College of Medicine, One Baylor Plaza, Houston, TX 77030, U.S.A.; 4 Structural Biology Center, Biosciences Division, Argonne National Laboratory, 9700 Cass Avenue, Argonne, IL 60439, U.S.A.

**Keywords:** AAA proteins, crystallography, heat shock proteins, molecular chaperones, site-directed mutagenesis, Saccharomyces cerevisiae

## Abstract

The ring-forming Hsp104 ATPase cooperates with Hsp70 and Hsp40 molecular chaperones to rescue stress-damaged proteins from both amorphous and amyloid-forming aggregates. The ability to do so relies upon pore loops present in the first ATP-binding domain (AAA-1; loop-1 and loop-2 ) and in the second ATP-binding domain (AAA-2; loop-3) of Hsp104, which face the protein translocating channel and couple ATP-driven changes in pore loop conformation to substrate translocation. A hallmark of loop-1 and loop-3 is an invariable and mutational sensitive aromatic amino acid (Tyr^257^ and Tyr^662^) involved in substrate binding. However, the role of conserved aliphatic residues (Lys^256^, Lys^258^, and Val^663^) flanking the pore loop tyrosines, and the function of loop-2 in protein disaggregation has not been investigated. Here we present the crystal structure of an N-terminal fragment of *Saccharomyces cerevisiae* Hsp104 exhibiting molecular interactions involving both AAA-1 pore loops, which resemble contacts with bound substrate. Corroborated by biochemical experiments and functional studies in yeast, we show that aliphatic residues flanking Tyr^257^ and Tyr^662^ are equally important for substrate interaction, and abolish Hsp104 function when mutated to glycine. Unexpectedly, we find that loop-2 is sensitive to aspartate substitutions that impair Hsp104 function and abolish protein disaggregation when loop-2 is replaced by four aspartate residues. Our observations suggest that Hsp104 pore loops have non-overlapping functions in protein disaggregation and together coordinate substrate binding, unfolding, and translocation through the Hsp104 hexamer.

## Introduction

Members of the ring-forming Hsp104/ClpB family of ATP-driven molecular chaperones are the principle protein disaggregases in fungi (Hsp104), plants (Hsp101), and eubacteria (ClpB) [1–3]. Interestingly, Hsp104 homologs are not found in animal cells [4], making members of this family a potential antimicrobial drug target. To rescue stress-damaged proteins from an aggregated state, Hsp104/ClpB disaggregases must cooperate with the cognate Hsp70/DnaK system, consisting of Hsp70 and Hsp40 in yeast and DnaK–DnaJ–GrpE in eubacteria, to form a potent bi-chaperone system. However, unlike the bacterial bi-chaperone system, a nucleotide-exchange factor such as yeast Sse1 [5] is not required for Hsp104-dependent protein disaggregation *in vitro* [6], but was shown to enhance its potency in yeast [7].

At the molecular level, *Saccharomyces cerevisiae* Hsp104 forms a homo-hexamer that is stabilized by adenine nucleotides [8–10]. Each Hsp104 monomer features two ATP-binding domains, termed AAA-1 and AAA-2, in addition to an N-terminal (N) domain and a coiled-coil motif that mediates the physical interaction with Hsp70 [11,12]. It is now widely accepted that Hsp104 facilitates the unfolding of aggregated proteins and the threading of substrate through the protein translocation channel analogous to ATP-dependent Clp proteases [13,14]. However, it remains unknown whether substrate unfolding and threading represent concerted or mechanistically distinct events. Amongst the Hsp104 domains, the functional role of the N domain is perhaps most perplexing. Although dispensable for protein disaggregation *in vitro* and *in vivo* [15–17], the N domain is essential for yeast prion dissolution [18] and curing by Hsp104 overexpression [15]. Consequently, how Hsp104 recognizes substrates and recovers stress-damaged proteins from protein aggregates has been a matter of considerable debate. Southworth and colleagues recently reported the high-resolution cryoEM structure of yeast Hsp104 bound to casein [19], an unstructured phosphoprotein that, unlike native substrates, is processed in a nucleotide-independent manner [20]. The structure confirmed a role for the conserved loop-1 and loop-3 tyrosines contacting the unfolded polypeptide, which is corroborated by an analogous cryoEM study of the bacterial homolog, ClpB [21]. However, the role of conserved aliphatic pore loop residues and the importance of loop-2 in protein disaggregation has not been investigated.

Here we present the X-ray structure of a *S. cerevisiae* Hsp104 fragment (Hsp104_1-360_) determined from a new crystal form featuring three independent copies of Hsp104_1-360_ in the crystallographic asymmetric unit. As each monomer has a different crystal-packing environment, consistent stereochemical features are inherent to the structure and independent of the crystal lattice. We find that both loop-1 and loop-2 form molecular interactions that resemble contacts with bound substrate. Although the structure of the physiological ring assembly was not determined, we show that the aliphatic side chains of Lys^256^ and Lys^258^ (loop-1) and Val^663^ (loop-3) flanking the conserved pore loop tyrosines are also involved in substrate interaction, and abolish Hsp104 function when mutated to glycine. Furthermore, our structure also suggests a previously unknown role for loop-2 in Hsp104 function. Although loop-2 shows only a small defect when all four residues are mutated to glycine or alanine, we find that loop-2 is sensitive to substitutions with aspartate. Notably, substituting loop-2 with four aspartates abolishes protein disaggregation *in vitro* and severely impairs thermotolerance development *in vivo*. Taken together, our observations suggest that loop-1 and loop-2 have distinct mechanical functions, and cooperate with loop-3 to facilitate the recovery of stress-damaged protein from aggregates.

## Experimental

### Protein expression and purification

*S. cerevisiae* Hsp104_Y257A_ and Hsp104_Y662A_ were generated by QuikChange site-directed mutagenesis (Agilent). All other Hsp104 pore-loop mutants were generated by overlap extension PCR followed by cassette mutagenesis. Hsp104 and its mutants were cloned into the pProEX-HTb vector (Invitrogen), which adds a tobacco etch virus protease cleavable N-terminal His_6_-tag, and were overexpressed in *Escherichia coli* BL21-CodonPlus (DE3)-RIL cells (Agilent) by isopropyl β-d-thiogalactopyranoside induction. Proteins were purified from cleared lysates by affinity chromatography on nickel-nitrilotriacetic acid (Ni-NTA) agarose column (Qiagen) and eluted in 25 mM Tris/HCl pH 7.5, 300 mM NaCl, 5% glycerol, and 5 mM β-mercaptoethanol containing 300 mM imidazole, or in TBS using a 20–800 mM imidazole gradient (Hsp104_1-360_). The N-terminal His_6_-tag was cleaved off and removed by reapplying the protein to a Ni-NTA agarose column. Hsp104_1-360_ was further purified by negative binding to an anion-exchange column followed by binding to a Mono-S column (GE Healthcare). His_6_-Ydj1 and His_6_-Hsp70 were purified as described [22].

### Size-exclusion chromatography

Full-length Hsp104 and Hsp104 mutant proteins were further purified by size-exclusion chromatography on a Superdex 200 10/300 GL column (GE Healthcare) pre-equilibrated in 25 mM Tris/HCl pH 7.5, 150 mM NaCl, 5% glycerol, and 1 mM DTT. Size-exclusion chromatography was also used to determine the oligomeric state of Hsp104 and Hsp104 mutants. Hexamers were isolated and used for subsequent ATPase activity measurements and coupled chaperone assays.

### Crystal structure determination

Crystals of Hsp104_1-360_ were grown by the hanging drop vapor diffusion method at 4°C by mixing 2 µl of protein solution (20 mg/ml) with 2 µl of reservoir solution containing 25% PEG 4000 (w/v), 50 mM Tris/HCl pH 8.5, and 20 mM ammonium citrate. Data were collected and processed using the HKL2000 software package [23] (Supplementary Table S1). The crystal structure of Hsp104_1-360_ was determined by molecular replacement using MOLREP [24] with Protein Data Bank (PDB) accession 6AMN as search model [25]. Two molecules of Hsp104_1-360_ were found. The calculated map revealed the location of a third molecule in the asymmetric unit, and the two domains of the third molecule were manually placed. After rigid body refinement, the N and AAA-1_large_ domains were connected in each molecule. Cycles of rebuilding and refinement were carried out using COOT [26] and PHENIX [27], respectively. Atomic coordinates and structure factors have been deposited in the PDB with the accession number 5WBW. Protein domain motions were analyzed using DynDom [28].

### ATPase activity assay

Hsp104 and variants (0.5 µM monomer) were incubated with 2 mM ATP at 22°C for 15 min. The amount of released inorganic phosphate was measured using the Malachite Green assay [29].

### Coupled chaperone assay

Firefly luciferase (FFL; 10 µM) was denatured in 7 M urea in refolding buffer (25 mM HEPES-KOH pH 7.5, 150 mM potassium acetate, 10 mM magnesium acetate, and 10 mM DTT) for 30 min at 22°C, then diluted 125-fold in refolding buffer containing the bi-chaperone system (1 μM Hsp104, 1 μM hHsp70, 1 μM Ydj1), 5 mM ATP, and an ATP-regenerating system consisting of 25 mM phosphoenolpyruvate and 2 μM pyruvate kinase. β-galactosidase (β-gal; 0.4 µM) was heat aggregated in refolding buffer for 40 min at 59°C and mixed (0.2 µM final concentration) with the bi-chaperone system (1 µM each) together with 4 mM ATP, 20 mM phosphoenolpyruvate, and 2 μM pyruvate kinase. Recovered enzymatic activities were measured after 120 min (FFL) and 360 min (β-gal) as described [30].

### Thermotolerance assay

Hsp104 loop-1 and loop-2 mutants were generated by excising an EcoRI-BglII fragment featuring the desired mutation and swapping it into pYS104 containing *S. cerevisiae* Hsp104 wild-type under control of the *Hsp104* promoter. Hsp104 loop-3 mutants were generated by cassette mutagenesis. Plasmids expressing wild-type and mutant Hsp104 were transformed into *S. cerevisiae* OT46 (Δ*hsp104*) and screened on synthetic defined growth medium without uracil (SD-Ura) plates [31]. Yeast cells were diluted to 0.1 *D_600_* from overnight cultures, grown for 2.5 h at 25°C in yeast extract peptone dextrose (YPD) medium and divided into two sets. One set was treated by heat-shock at 50°C for 15 min (basal thermotolerance), while the other set was incubated at 37°C for 30 min to induce heat-shock protein synthesis prior to heat-shock (induced thermotolerance). Cells were heat-shocked and immediately chilled on ice. Five microliters of ten-fold serial dilutions were dropped on to YPD plates. Viability was scored after 2 days of incubation at 30°C.

### Subunit mixing experiments

Hsp104 and mutant proteins were mixed at different ratios to achieve the indicated subunit composition in the hexamer, while keeping the total protein concentration at 10 µM. Mixtures were incubated at 22°C for 20 min to allow for subunit exchange. For urea-denatured FFL, Hsp104 hetero-hexamers were diluted ten-fold with refolding buffer containing 1 µM Hsp70 and Hsp40. For heat-aggregated β-gal, 0.3 µM of the bi-chaperone system with Hsp104 hetero-hexamers was used. As control, Hsp104, Hsp70, and Hsp40 chaperones were diluted with refolding buffer keeping their stoichiometric ratio constant. Coupled chaperone assays were performed in the presence of ATP and an ATP regenerating system as described above.

## Results

### Crystal structure of Hsp104_1-360_

Yeast Hsp104 is a protein disaggregase that is functionally conserved with bacterial ClpB [32]. The crystal structure of *Chaetomium thermophilum* Hsp104 confirmed that Hsp104 and ClpB are also structurally conserved [33]. However, the atomic structures of the N- and C-terminal domains of *C. thermophilum* Hsp104 could not be modeled despite being part of the crystallized construct. Here we present the orthorhombic crystal structure of an N-terminal fragment of *S. cerevisiae* Hsp104 (Hsp104_1-360_) comprising the N domain (residues 4–164), the AAA-1_large_ domain (residues 165–341), and the first α-helix of the AAA-1_small_ domain (residues 345–356). We did not observe any unaccounted electron density that could be attributed to a bound nucleotide, even when 5 mM nucleotide (ADPNP or ADP) was added for crystallization, indicating that Hsp104_1-360_ was crystallized in the nucleotide-free state. The structure of Hsp104_1-360_ was determined by molecular replacement and was refined to a resolution of 2.6 Å (Supplementary Table S1). The crystal structure consists of three Hsp104_1-360_ monomers (mol 1, mol 2, and mol 3) that are structurally independent and in different physicochemical environments, which allows the identification of common structural features that may be of functional importance.

### Crystal structure of Hsp104_1-360_ confirms the high *en bloc* mobility of the N domain

**Figure 1:**
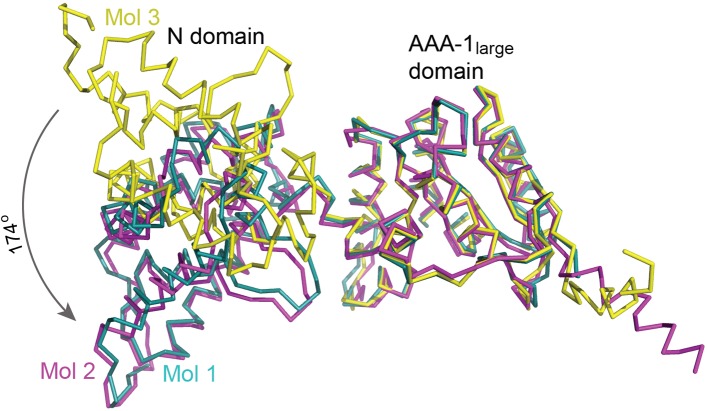
Crystal structure of Hsp104_1-360_ Superposition of the three Hsp104_1-360_ monomers shows the *en bloc* mobility of the N domains. Atomic structures were superimposed through their AAA-1_large_ domain. Molecule 1 (mol 1) is shown in teal, molecule 2 (mol 2) in magenta, and molecule 3 (mol 3) in yellow.

The atomic structures of the N and AAA-1_large_ domains alone are nearly identical amongst the three Hsp104_1-360_ molecules and superimpose pairwise with an RMSD of only 0.41 ± 0.05 Å (N domain) and 0.72 ± 0.22 Å (AAA-1_large_). In addition, the three Hsp104_1-360_ molecules superimpose pairwise with the hexagonal crystal structure of one Hsp104_1-360_ monomer (PDB: 6AMN) [25] with an RMSD of 0.47 ± 0.01 Å (N domain) and 0.75 ± 0.01 Å (AAA-1_large_), and with the crystal structure of the isolated *S. cerevisiae* Hsp104 N domain (PDB: 5U2U) [34] with an RMSD of 0.50 ± 0.05Å calculated over all atoms. Superimposing the crystal structures of the complete Hsp104_1-360_ fragment through their AAA-1_large_ domain shows that the orientation of the N domain seen in mol 1 and mol 2 is rotated by 172–174° relative to that in mol 3 (Figure 1) with residues 161–165 making up the hinge region. Interestingly, different N domain conformations are also observed in the fitted cryoEM structures of Hsp104 hexamers, with mol 1 and 2 matching the N domain conformation of the C subunit of the open conformation (PDB: 5KNE-C) [10] and the A, C, and E subunits of the closed conformation of Hsp104 with casein bound (PDB: 5VY9-A/C/E) [19]. Mol 3 matches the F subunit of the casein-bound, closed structure (PDB: 5VY9-F) [19]. It is noteworthy that the N domain conformation of the Hsp104_1-360_ monomer in the hexagonal crystal form (mol 4) [25] differs from the other three conformations presented here and matches the N domain conformation of the D subunit of the casein-bound, closed structure (PDB: 5VY9-D) [19]. Together, these findings indicate that the high *en bloc* mobility of the N domain observed in our crystal structure is likely to be of functional importance, and is also observed in physiologically relevant structures of Hsp104 hexamers.

The AAA-1_large_ domain shares the canonical α/β-fold of related AAA+ ATPases determined in their hexamer assembly [35–38], and superposes with the crystal structure of the isolated AAA-1_large_ domain of *E. coli* ClpB (PDB: 1JBK) [39] with an RMSD of 1.20 ± 0.11 Å calculated over their Cα atoms. However, unlike previously determined crystal structures, we observed both pore loops in our structure. The first AAA-1 pore loop (loop-1), comprising residues 253–259, is seen in all three monomers, and loop-2, comprising residues 291–294, is ordered in two molecules. Loop-1 features the conserved Tyr^257^ that is sensitive to alanine mutation in Hsp104 [40], and can be site specifically cross-linked to substrate-mimicking peptides in ClpB [41]. A pore-facing tyrosine or phenylalanine is also found in many other AAA+ machines involved in protein quality control [42], and support a key role for Tyr^257^ in substrate binding, translocation, or both. Although no corresponding aromatic residue is found in loop-2 that features only two non-glycine residues, loop-2 was shown to be sensitive to mutation that impairs the protein unfolding activity of bacterial ClpA [43].

### Hsp104_1-360_ monomer contacts resemble interaction with substrate

**Figure 2:**
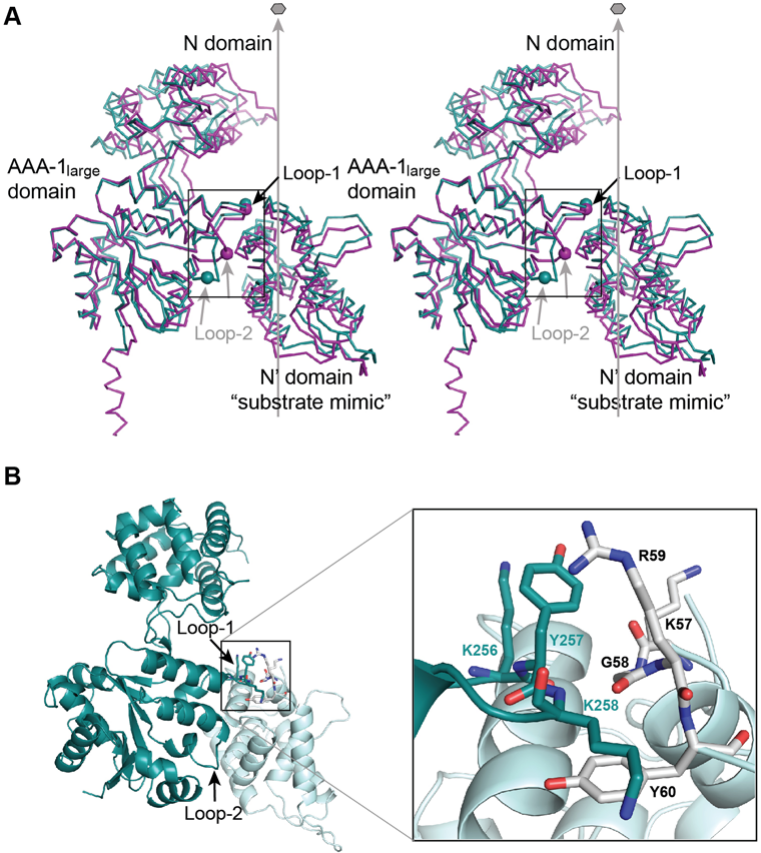
Stereochemistry of molecular interactions between AAA-1 pore loops and a substrate mimic (**A**) Stereoview of the molecular contacts between loop-1/-2 of mol 1 (teal) and mol 2 (magenta) and the N′ domain of an NCS-related neighboring molecule that mimics a bound substrate. The Cα positions of Tyr^257^ (loop-1) and of Asn^292^ (loop-2) are shown as spheres. The protein-translocating channel that traverses the Hsp104 hexamer is indicated by the six-fold axis. The figure shows that loop-2 adopts an ‘up’ and ‘down’ configuration in the crystal, which may resemble ATP-driven conformations associated with substrate translocation. (**B**) Molecular interface between loop-1 and the bound substrate mimic. Loop-1 residues are shown in teal and N′ domain residues in gray. The inset shows a close-up view of the same interface.

In our structure, Tyr^257^ makes contact with the N domain of a neighboring, non-crystallographic symmetry (NCS) related molecule (Figure 2A,B). Tyr^257^ is flanked by Lys^256^ and Lys^258^ that contributes to the protein–protein interface made up of hydrophobic contacts between aliphatic and aromatic side chains of loop-1 and N domain residues, Arg^59^ and Tyr^60^, with additional contributions from the main chain of residues Lys^57^, Gly^58^, and Arg^59^ (Figure 2B). The aforementioned hydrophobic contacts are reminiscent of a chaperone–substrate interaction, and are observed in two out of three molecules (Figure 2A). However, neither the hydroxyl group of the Tyr^257^ side chain nor the ε-amino group of Lys^258^ contributes binding energy (Figure 2B). In addition, the aliphatic side chain of Lys^256^ forms a stacking interaction with the Tyr^257^ side chain, which may orient Tyr^257^, while the ε-amino group of Lys^256^ makes an ionic interaction with the Glu^146^ side chain of a neighboring molecule. Our observations support a functional role for Lys^256^ and Lys^258^ in substrate interaction, and provide an explanation why aromatic residues, such as tryptophan and phenylalanine, can substitute for conserved pore loop tyrosines without marked loss of Hsp104 function [40].

Unlike loop-1, loop-2 is less well ordered, which prevented us from modeling side chains. In our structure, loop-2 adopts two distinct conformations in an ‘up’ and ‘down’ configuration when viewed along the six-fold axis of the Hsp104 hexamer (Figures 2A and 4A). Residue 292 (asparagine) that is non-conserved in ClpA/B proteins, is in van der Waals contact with the N domain of an NCS-related, neighboring molecule (Lys^131^), resembling an interaction with substrate. In support of a functional role for Asn^292^ in substrate binding, it was shown that mutating the analogous residue in bacterial ClpA (Ala^293^) from alanine to aspartate abolished binding and translocation of an unfolded model substrate [43]. It is tempting to speculate that the ‘up’ and ‘down’ configurations of loop-2 may represent conformations associated with protein unfolding or substrate translocation through the central channel of the Hsp104 hexamer, and is subject to future investigations.

### The hydrophobicity but not aromaticity of loop-1 is crucial for protein interaction

**Figure 3:**
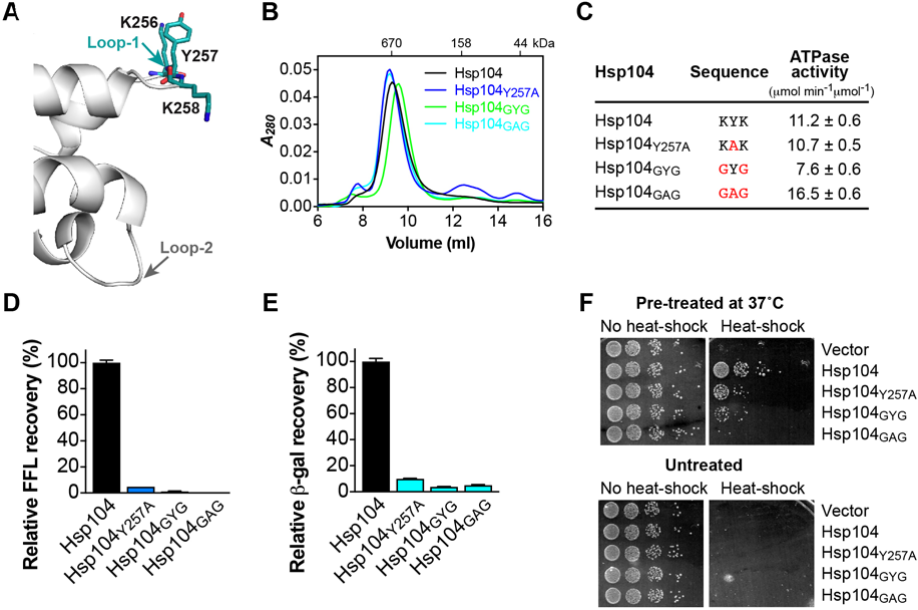
Loop-1 mediates protein–protein interactions essential to Hsp104 function (**A**) Ribbon diagram showing the location of loop-1 relative to loop-2 (gray). Residues of the ^256^Lys-Tyr-Lys^258^ tripeptide motif that mediate substrate interaction are colored and shown as stick model. (**B**) Size-exclusion chromatograms of Hsp104 and loop-1 mutants. (**C**) ATPase activities of loop-1 mutants. Mutated residues are shown in red. (**D**,**E**) Coupled chaperone assay showing the relative recovery of enzymatic activity by loop-1 mutants in the presence of the Hsp70 chaperone system with (D) chemically denatured FFL and (E) heat-aggregated β-gal as substrate. Means of three independent measurements ± S.D. are shown. (**F**) Induced (top) and basal (bottom) thermotolerance of Δ*hsp104* yeast expressing the empty vector, Hsp104, or loop-1 mutants.

The functional importance of conserved pore loop tyrosines in Clp/Hsp100 proteins is well established [41,43–45]. It was shown more recently that Tyr^257^ of Hsp104 mediates binding of an unstructured polypeptide [19]. Consistent with a role in substrate interaction, Tyr^257^ is sensitive to mutation that severely impaired but, interestingly, did not abolish Hsp104 function [40]. The latter suggests that other pore loop residues must also contribute toward substrate binding. The crystal structure of Hsp104_1-360_ revealed a previously unobserved specific interface between loop-1 and the N domain of a neighboring, NCS-related molecule involving the side chains of Lys^256^ and Lys^258^ in addition to Tyr^257^ (Figures 2B and 3A), contrasting the proposed role of Lys^256^ and Lys^258^ in stabilizing the hexamer assembly [19].

To our knowledge, the importance of conserved aliphatic residues flanking Tyr^257^ has not been investigated previously. We therefore mutated Lys^256^ and Lys^258^ to glycine (Hsp104_GYG_) and compared the activity of Hsp104_GYG_ with Hsp104_Y257A_ that is functionally impaired. As expected, all of our loop-1 mutants assemble into hexamers (Figure 3B) and are functional ATPases (Figure 3C). Strikingly, we find that replacing Lys^256^ and Lys^258^ with glycine severely impaired Hsp104 function *in vitro* (Figure 3D,E) and *in vivo* (Figure 3F), even more so than Hsp104_Y257A_ (Figure 3D–F). Because a hexamer ring assembly is a prerequisite for ATP hydrolysis [30], the ability of Hsp104_GYG_ to hydrolyze ATP argues against a role of Lys^256^ and Lys^258^ in the formation of hexamers or stabilizing the oligomer assembly. Combining the Lys^256^, Tyr^257^, and Lys^258^ mutations (Hsp104_GAG_) completely abolished Hsp104’s ability to disaggregate chemically denatured FFL *in vitro* (Figure 3D) and its ability to acquire thermotolerance *in vivo* (Figure 3F, compared Hsp104_GAG_ with vector control). Taken together, our observations support a role for Lys^256^ and Lys^258^ in substrate interaction that is abolished when the ^256^Lys-Tyr-Lys^258^ tripeptide is mutated to glycine and alanine, respectively.

### Loop-2 is sensitive to aspartate substitutions

**Figure 4:**
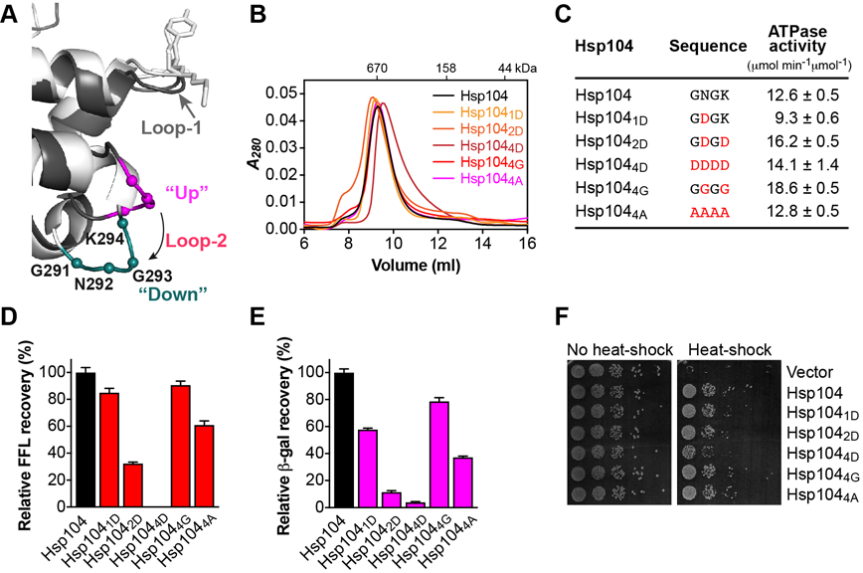
Loop-2 is sensitive to mutation and potentially promotes substrate unfolding (**A**) Ribbon diagram generated by superimposing the AAA-1_large_ domain of mol 1 (light gray) and mol 2 (dark gray), showing the location of loop-2 (magenta/teal) relative to loop-1 (gray). Loop-2 adopts an ‘up’ (magenta) and ‘down’ configuration (teal) that may mimic conformations associated with substrate unfolding. Cα positions of loop-2 residues are shown as spheres. (**B**) Size-exclusion chromatograms of Hsp104 and loop-2 mutants. (**C**) ATPase activities of loop-2 mutants. Mutated residues are shown in red. (**D**,**E**) Coupled chaperone assay showing the relative recovery of enzymatic activity by loop-2 mutants in the presence of the Hsp70 chaperone system with (D) chemically denatured FFL and (E) heat-aggregated β-gal as substrate. Means of three independent measurements ± S.D. are shown. (**F**) Induced thermotolerance of Δ*hsp104* yeast expressing the empty vector, Hsp104, or loop-2 mutants.

The crystal structure of Hsp104_1-360_ showed that loop-2 adopts an ‘up’ (mol 2) and ‘down’ (mol 1) configuration with the tip of loop-2 making van der Waals contact with a neighboring, NCS-related molecule (Figures 2A and 4A). The latter is suggestive of a substrate interaction and, when taken together, reminiscent of an interaction with substrate that is being translocated down the axial channel. Because a functional role for loop-2 in substrate binding or translocation has not been demonstrated previously for Hsp104/ClpB, we asked whether loop-2 is sensitive to mutation that would impact Hsp104 function.

Loop-2 is considerably shorter than other pore loops and consists of only four amino acid residues of sequence ^291^Gly-Asn-Gly-Lys^294^ (Figure 4A). It was previously reported that mutating the equivalent residue of Asn^292^ of *E. coli* ClpA (Ala^293^) to aspartate abolished ClpA function [43]. We therefore asked whether introducing one or more aspartates into loop-2 would have a similar impact on Hsp104 function. As anticipated, loop-2 mutants assemble into hexamers (Figure 4B and Supplementary Figure S1) and are functional ATPases (Figure 4C). We note that the Hsp104_4D_ hexamer is right shifted in the absence of nucleotide (Figure 4B), but elutes at the expected position in the presence of ATPγS (Supplementary Figure S1). Furthermore, we found that the ATPase activity of Hsp104_4D_ is similar to Hsp104 wild-type (Figure 4C), indicating no structural perturbations. Yet, replacing Asn^292^ with aspartate (Hsp104_1D_) reduced the recovery of chemically denatured FFL by the bi-chaperone system by 15% (Figure 4D). Introducing a second aspartate (Hsp104_2D_) further reduced protein disaggregation by 68%, and substituting all four residues (Hsp104_4D_) completely abolished Hsp104-dependent protein disaggregation *in vitro* (Figure 4D). The observed loss-of-function is specific to Hsp104_4D_ because loop-2 variants featuring either four alanine (Hsp104_4A_) or four glycine residues (Hsp104_4G_) cooperate with the Hsp70 system in protein disaggregation (Figure 4D). Similar results were also obtained with heat-aggregated β-gal as the model substrate arguing against a substrate-specific defect (Figure 4E). Consistent with our *in vitro* observations, loop-2 mutants are also impaired *in vivo*, with Hsp104_4D_ showing the largest defect in thermotolerance development (Figure 4F).

Taken together, our observations suggest that loop-2 is sensitive to aspartate substitutions that severely impair Hsp104 function when loop-2 is replaced by four aspartates. The inability of Hsp104_4D_ to recover functional protein from aggregates could not be overcome by loop-1 (Figure 4D,E), nor could loop-2 rescue loop-1 loss-of-function mutants (Figure 3D,E), suggesting that loop-1 and loop-2 have distinct, non-overlapping roles in protein disaggregation. Although the exact nature of the functional defect of loop-2 mutants remains unclear, we speculate that the aspartate substitutions may have interfered with substrate interaction. Furthermore, the apparent lack of specificity observed with Hsp104_4G_ that remains fully functional (Figure 4D,E) contrasts the proposed role of loop-1 as a substrate anchor that facilitates a tight interaction with substrate and is sensitive to glycine/alanine substitutions. Thus, our observations could be indicative of a more mechanical function of loop-2 in protein unfolding or translocation, which does not require a tight grip on substrate.

### Loop-3 is essential for protein disaggregation

**Figure 5:**
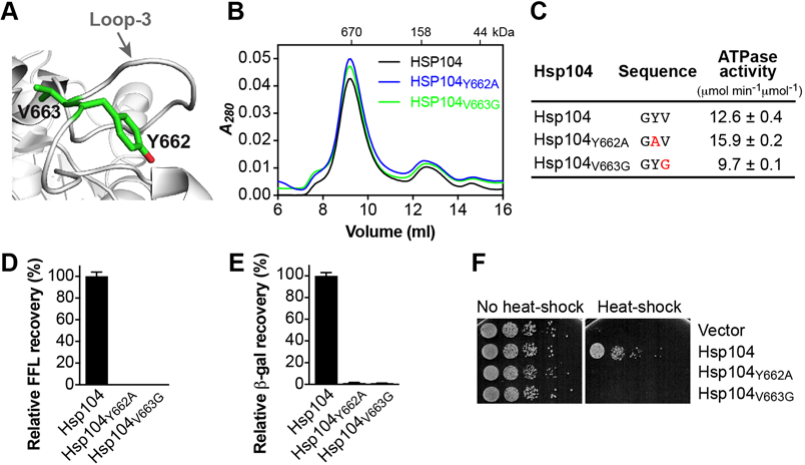
Loop-3 is critical for substrate binding and translocation through the distal ring (**A**) Ribbon diagram of *C. thermophilum* Hsp104 [33] showing the location of loop-3 with the side chains of Tyr^662^ and Val^663^ represented as green stick model. (**B**) Size-exclusion chromatograms of Hsp104 and loop-3 mutants. (**C**) ATPase activities of loop-3 mutants. Mutated residues are shown in red. (**D**,**E**) Coupled chaperone assay showing the relative recovery of enzymatic activity by loop-3 mutants in the presence of the Hsp70 chaperone system with (D) chemically denatured FFL and (E) heat-aggregated β-gal as substrate. Means of three independent measurements ± S.D. are shown. (**F**) Induced thermotolerance with Δ*hsp104* yeast cells expressing the empty vector, Hsp104, or loop-3 mutants.

Loop-3 features a conserved aromatic amino acid (Tyr^662^) that is essential for substrate interaction *in vitro* and *in vivo* [40] (Figure 5A–F). Tyr^662^ is preceded by glycine or a small aliphatic residue and is followed by a hydrophobic amino acid (Ψ) and glycine, giving rise to a (Gly)-Tyr-Ψ-Gly motif. The latter is reminiscent to the Ψ-Tyr-Ψ motif of loop-1, which impaired Hsp104 chaperone function when Ψ is replaced with glycine (Figure 3D–F). In Hsp104, Tyr^662^ is followed by Val^663^ that shares an aliphatic side chain with Lys^258^. It was previously shown that the equivalent valine in heat-shock locus U (HslU) (Val^92^) is insensitive to isoleucine, alanine, and serine substitutions, but abolishes protein unfolding and translocation when mutated to phenylalanine or cysteine [46]. Similar observations were also made in bacterial ClpX with observed levels of impairment dependent on the substrate [44]. It is interesting to note that all of the aforementioned Clp/Hsp100 variants featuring aliphatic side chain substitutions, besides cysteine that is sensitive to oxidation, appear to be functional. We therefore asked whether replacing Val^663^ with glycine that lacks an aliphatic side chain would impact protein disaggregation. Strikingly, we found that protein disaggregation by Hsp104_V663G_ was completely abolished *in vitro* (Figure 5D,E) and *in vivo* (Figure 5F), despite featuring a functional Tyr^662^. The observed defect was not due to an inability to self-assemble or lack of ATPase activity, which was similar to Hsp104 wild-type (Figure 5B,C). The sequence specificity and location of loop-3 near the distal end of the protein translocating channel support an essential role of loop-3 in polypeptide binding and translocation. However, it is not the conservation of Tyr^662^ *per se* but the hydrophobicity of loop-3 that is essential to Hsp104 function.

### Loop-1 and loop-2 cooperate in initial protein binding and unfolding

Our *in vitro* and *in vivo* experiments show that loss-of-function mutations of either loop-1 or loop-2 can abolish protein disaggregation, suggesting distinct, non-overlapping functions of AAA-1 pore loops. While Tyr^257^ is critically important for substrate binding [19,40,41], our structure further extends the substrate-binding interaction to the ^256^Lys-Tyr-Lys^258^ tripeptide (Figure 2B). Interestingly, both Hsp104_KAK_ (i.e. Hsp104_Y257A_) and Hsp104_GYG_ retain some chaperone activity and mutation of all three residues is required to abolish Hsp104 function (Figure 3D,F). It is worth noting that the proposed hydrophobic interaction between loop-1 and substrate is consistent with the prevailing notion of molecular chaperones in recognizing exposed hydrophobic residues to discriminate between folded and unfolded protein conformers.

**Figure 6:**
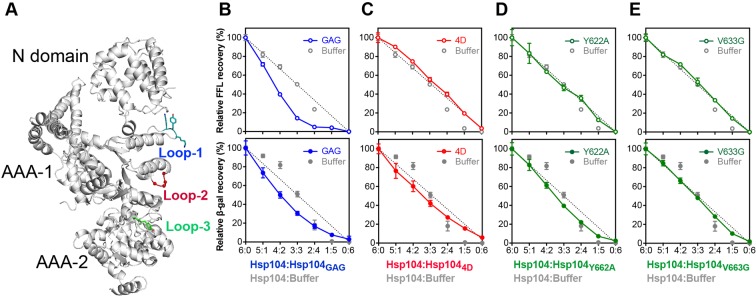
Protein disaggregation by Hsp104 hetero-oligomers composed of active and inactive pore loop mutant subunits (**A**) Ribbon diagram of a composite model of an Hsp104 monomer generated by superposing the AAA-1_large_ domain of yeast Hsp104_1-360_ on to the crystal structure of *C. thermophilum* Hsp104 [33]. Channel facing loops are colored in teal (loop-1), red (loop-2), and green (loop-3). (**B**–**E**) Relative recoveries of FFL and β-gal activities by Hsp104 hetero-oligomers composed of wild-type and pore loop mutant subunits in the presence of the Hsp70 chaperone system. Means of three independent measurements ± S.D. are shown. The dashed line represents the linear decrease expected if the activity of the Hsp104 hexamer is proportional to the number of wild-type subunits present. Buffer only dilutions are also shown. (B) Hsp104:Hsp104_GAG_ (loop-1), (C) Hsp104:Hsp104_4D_ (loop-2), (D) Hsp104:Hsp104_Y662A_ (loop-3), and (E) Hsp104:Hsp104_V663G_ (loop-3).

The recently reported cryoEM structure of a casein-bound Hsp104 hexamer supports a threading mechanism down the central channel, necessitating cooperative interactions between adjacent subunits [19]. To determine whether pore loops of neighboring Hsp104 subunits cooperate in protein disaggregation, we used a subunit mixing assay [22] to monitor protein disaggregation by Hsp104 hexamers composed of active and inactive mutant subunits. We note that all three pore loops have distinct locations within one subunit, and are not in direct contact (Figure 6A). However, we do not rule out contacts with loops in neighboring subunits as previously proposed [19]. Figure 6B shows that protein disaggregation by Hsp104:Hsp104_GAG_ hetero-hexamers together with the Hsp70 system was substantially impaired in the presence of only one inactive Hsp104_GAG_ subunit. The latter suggests strong cooperativity between subunits and lend support for substrate handover between loop-1 of neighboring AAA-1 domains. A substrate handover mechanism is supported by the recent cryoEM structure of a casein-bound Hsp104 hexamer revealing direct contacts of loop-1 residues from neighboring subunits with the unfolded polypeptide [19]. A more complex pattern emerges when performing the subunit-mixing experiment with Hsp104 hexamers composed of wild-type and inactive loop-2 mutant subunits (Hsp104_4D_) (Figure 6C). Both cooperative and probabilistic interactions are observed depending on the nature of the substrate (Figure 6C). While a cooperative interaction between subunits was observed with heat-aggregated β-gal, a near linear decline was seen with chemically denatured FFL, indicating a probabilistic mechanism. Although it may seem that Hsp104 uses distinct *modi operandi*, we reasoned that only heat-aggregated and amyloid-forming substrates that are characterized by a stable secondary and/or tertiary structure [47,48] may require an additional protein unfolding step prior to substrate translocation. Because loop-2 mutants featuring either four glycines (Hsp104_4G_) or four alanines (Hsp104_4A_) are functional, and cooperate with Hsp70 and Hsp40 chaperones in protein disaggregation (Figure 4D–F), the observed defect of Hsp104_4D_ may be indicative of a mechanical function in protein unfolding or translocation, and is reflected in the nature of the substrate used.

Taken together, we propose that unstructured model substrates, such as casein and chemically unfolded FFL that are tethered to loop-1, do not require mechanical unfolding prior to substrate translocation. On the other hand, heat-aggregated substrates rely on an additional mechanical unfolding step that is dependent on cooperative interactions between loop-2 from neighboring subunits to exert a stronger pulling force.

### Loop-3 is essential for substrate translocation through the distal ring

The distinct cooperative and probabilistic mechanisms observed with hetero-hexamers composed of active and inactive loop-2 subunits were intriguing. We therefore performed subunit-mixing experiments with active and inactive loop-3 variants (Hsp104_Y662A_ and Hsp104_V633G_). In agreement with the literature [49], we observed a near linear decrease in FFL reactivation as the number of Hsp104_Y662A_ subunits increased (Figure 6D). We note that the pattern differs somewhat for heat-aggregated β-gal as seen with loop-2 mutant hetero-hexamers (Figure 6C). Mixing Hsp104 with Hsp104_V633G_ yields hetero-hexamers that do not function cooperatively in protein disaggregation (Figure 6E). How can these differences be reconciled? Loop-3 is at the distal end of the protein-translocating channel where the unfolded polypeptide emerges from the Hsp104 hexamer. Because Tyr^662^ has previously been shown to bind polypeptides [40,45], Tyr^662^ may provide a substrate anchor with Val^663^ adding binding energy by contacting the unfolded polypeptide. We speculate that the observed cooperativity of Hsp104:Hsp104_Y662A_ hetero-hexamers with heat-aggregated β-gal compared with chemically denatured FFL might be the result of an additional protein unfolding step needed for native Hsp104 substrates, such as those encountered during heat-stress, and is dispensable for non-native substrates, such as chemically unfolded FFL and casein that is inherently unstructured.

## Discussion

Hsp104 chaperones are protein disaggregases that recover functional protein from both amorphous and amyloid-forming aggregates. Seminal discoveries from different laboratories have provided key insights into the protein disaggregation mechanism by the Hsp104 bi-chaperone system [1,2]. It is now widely accepted that the Hsp70 chaperone system targets Hsp104 to protein aggregates *in vivo* [17,50] from which Hsp104 extracts one or more polypeptides. The substrate is threaded through the Hsp104 hexamer [45,51], resulting in protein unfolding. We propose that pore loop-1 facilitates the interaction with substrate at the proximal end, providing an anchoring point for initial substrate binding.

Our crystal structure of Hsp104_1-360_ revealed an interaction between loop-1 and an NCS-related, neighboring molecule which mimics an interaction with substrate and supports a functional role of loop-1 as an anchor for initial substrate binding. Consistent with such a role, replacing the ^256^Lys-Tyr-Lys^258^ tripeptide motif of loop-1 in full-length Hsp104 with glycine and alanine, respectively, abolishes Hsp104 function without disrupting hexamer assembly. Unlike loop-1, the role of loop-2 is more complex. Loop-2 mutants are mostly functional in protein disaggregation, and Hsp104_4G_ that features four glycines instead of loop-2 is nearly as active as Hsp104 wild-type (Figure 4D–F). Yet, replacing loop-2 with four aspartates (Hsp104_4D_) abolishes protein disaggregation *in vitro* (Figure 4D,E) and impaired thermotolerance development *in vivo* (Figure 4F). Lack of sequence preference observed with Hsp104_4G_ and Hsp104_4A_ may be indicative of a more mechanical function of loop-2, such as what would be required to facilitate protein unfolding of heat-aggregated substrates. We speculate that loop-2, driven by ATP hydrolysis in the AAA-1 domain, moves from an ‘up’ to a ‘down’ position inside the central channel, exerting a mechanical pulling force on substrate bound to loop-1 in order to promote local unfolding. This unidirectional motion propels the substrate down the axial channel and, combined with substrate binding and pulling by loop-3, results in protein unfolding and translocation with the unfolded polypeptide emerging from the distal end.

Although our model inferred from the crystal structure of Hsp104_1-360_ is supported by both biochemical and functional studies *in vitro* and *in vivo*, Hsp104_1-360_ did not crystallize as a hexameric ring assembly. We therefore cannot exclude contacts between neighboring subunits, which may have impacted substrate binding. While our manuscript was under review, the cryoEM structures of a casein-bound Hsp104 hexamer was reported [19]. Although these structures provided a stereochemical framework to interpret our observations, any structural insight must be taken with caution because of the limited resolution and accuracy of these cryoEM reconstructions that remain to be confirmed biochemically. In the hexamer structure, both loop-1 and loop-3 make contact with the equivalent loops of neighboring subunits on both sides supporting a clockwise handover of the unfolded polypeptide when viewed top down. Furthermore, Lys^258^ of loop-1 interacts with residues of the neighboring subunit immediately following loop-2 (Asp^295^ and Asp^296^), which would be consistent with the complex pattern of substrate recovery observed with hexamers composed of active and inactive loop-2 subunits (Figure 6C). Taken together, we propose a coordinated interaction between pore loops with loop-1 facilitating protein binding, loop-2 potentially promoting protein unfolding by pulling down the substrate, and loop-3 mediating substrate translocation through the Hsp104 hexamer.

## Supporting information

**Supplementary Figure S1.** Analytical size-exclusion chromatograms of Hsp104 and Hsp1044D in the presence of nucleotide. Both Hsp104 (black) and Hsp104_4D_ (red) form hexamer assembles with ATPγS. Molecular weight standards are shown.

**Supplementary Table S1.** Data collection and refinement statistics Values in parenthesis are for the highest resolution shell.

## Abbreviations

β-gal: β-galactosidase
FFL: firefly luciferase
NCS: non-crystallographic symmetry
Ni-NTA: nickel-nitrilotriacetic acid
PDB: Protein Data Bank
SD-Ura: synthetic defined growth medium without uracil
YPD: yeast extract peptone dextrose
