# Supplementary material for: Structural determinants for protein unfolding and translocation by the Hsp104 protein disaggregase
Source: Biosci Rep. 2017 Dec 22;37(6):BSR20171399. doi: 10.1042/BSR20171399 (PMC5741831; doi:10.1042/BSR20171399)
Supplement: Supplementary file 1 [file bsr20171399_Supp1.pdf]

SUPPLEMENTARY MATERIAL

Structural Determinants for Protein Unfolding and Translocation by the Hsp104 Protein Disaggregase

Jungsoon Lee[1], Nuri Sung[1], Lythou Yeo[1], Changsoo Chang[4],

Sukyeong Lee[1*] and Francis T.F. Tsai[1,2,3*]

[1]Verna and Marrs McLean Department of Biochemistry and Molecular Biology, Baylor College of Medicine, One Baylor Plaza, Houston, Texas 77030, USA; [2]Department of Molecular and Cellular Biology, Baylor College of Medicine, One Baylor Plaza, Houston, Texas 77030, USA; [3]Department of Molecular Virology and Microbiology, Baylor College of Medicine, One Baylor Plaza, Houston, Texas 77030, USA and [4]Structural Biology Center, Biosciences Division, Argonne National Laboratory, 9700 Cass Avenue, Argonne, IL 60439, USA.

SUPPLEMENTARY METHOD

Analytical size-exclusion chromatography

Purified Hsp104 and $Hsp104_{4D}$ (15 μM monomer) were incubated with 5 mM ATPγS in refolding buffer (25 mM HEPES-KOH pH 7.5, 150 mM potassium acetate, 10 mM magnesium acetate, and 10 mM DTT) for 1 h on ice to allow self-assembly. Hsp104 and $Hsp104_{4D}$ oligomers were analyzed on a Superdex PC 3.2/30 column preequilibrated in 25 mM HEPES-KOH pH 7.5, 150 mM potassium acetate, 10 mM magnesium acetate, 1 mM DTT and 50 μM ATPγS. Molecular weight standards (Bio-Rad) are shown.

SUPPLEMENTARY TABLE

Supplementary Table S1. Data collection and refinement statistics

Values in parenthesis are for the highest resolution shell.

Data collection	
Wavelength (Å)	1.0396
Temperature (K)	93
Space group	$P\ 2_1 2_1 2_1$
a, b, c (Å)	66.56, 75.82, 235.65
α, β, γ (°)	90.0, 90.0, 90.0
Resolution range (Å)	49.04 - 2.59 (2.68 - 2.59)
Total No. of reflections	238,759
No. of unique reflections	36,873
Completeness (%)	97.3 (80.3)
Multiplicity	6.5 (3.9)
$\langle I/\sigma(I) \rangle$	16.5
R_{sym}[§]	0.058
Overall *B* factor from Wilson plot ($Å^2$)	64.54
Structure refinement	
Resolution range (Å)	46.04 - 2.60 (2.65 - 2.60)
Completeness (%)	97.76
No. of reflections, working/test set	36,797 / 2,587
Final R_{cryst}[¶]	0.2408 (0.3391)
Final R_{free}[¶]	0.2859 (0.4437)

No. of molecules in asymmetric unit	3
No. of non-H atoms	
Protein	7,864
Water	24
Total	7,888
R.m.s. deviations	
Bonds (Å)	0.002
Angles (°)	0.558
Average B factors ($Å^2$)	
Protein	97.85
Water	71.89
All atoms	97.77
Ramachandran plot	
Most favoured (%)	96.48
Allowed (%)	3.12
Outliers (%)	0.39

[§]$R_{sym} = \Sigma_{hkl} \Sigma_i |I_i(hkl) - <I(hkl)>| / \Sigma_{hkl} \Sigma_i I_i(hkl)$, where $<I(hkl)>$ is the mean of i observations $I_i(hkl)$ of reflection *hkl*.

[¶]R_{cryst} and $R_{free} = \Sigma_{hkl} ||F_{obs}| - |F_{calc}|| / \Sigma_{hkl} |F_{obs}|$, where F_{obs} and F_{calc} are the observed and calculated structure factors, respectively, calculated for recorded data (R_{cryst}) and for 7.03 % of the data omitted in refinement (R_{free}).

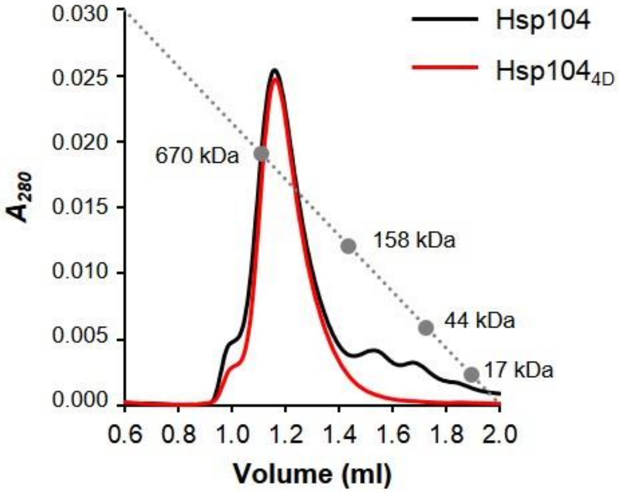

Supplementary Figure S1. Analytical size-exclusion chromatograms of Hsp104 and Hsp104$_{4D}$ in the presence of nucleotide. Both Hsp104 (black) and Hsp104$_{4D}$ (red) form hexamer assembles with ATPγS. Molecular weight standards are shown.
